# The effects of CYP2B6 inactivators on the metabolism of ciprofol

**DOI:** 10.1371/journal.pone.0307995

**Published:** 2024-07-29

**Authors:** Ming Lu, Xiaorui Zhang, Wenli Li, Xiangchen Li, Shan Li, Xiaoyu Yin, Zhiqing Zhang

**Affiliations:** 1 Department of Pharmacy, The Second Hospital of Hebei Medical University, Shijiazhuang, China; 2 Department of Pharmacy, Hebei General Hospital, Shijiazhuang, China; Manipal Academy of Higher Education, INDIA

## Abstract

Ciprofol is a novel short-acting intravenous anaesthetic developed in China that is mainly metabolized by cytochrome P450 2B6 (CYP2B6) and uridine diphosphate glucuronosyltransferase 1A9 (UGT1A9). Currently, insufficient evidence is available to support drug‒drug interactions between ciprofol and CYP2B6 inactivators. Here, we established a high-performance liquid chromatography-tandem mass spectrometry (HPLC-MS/MS) method to assess the concentration of ciprofol and investigated the effects of psoralen and clopidogrel on the metabolism of ciprofol in liver microsomes and rats. In rat and human liver microsomes, the median inhibitory concentration (*IC*_50_) values of psoralen were 63.31 μmol·L^-1^ and 34.05 μmol·L^-1^, respectively, showing mild inhibitory effects on ciprofol metabolism, whereas the *IC*_50_ values of clopidogrel were 6.380 μmol·L^-1^ and 2.565 μmol·L^-1^, respectively, with moderate inhibitory effects. SD rats were randomly divided into three groups: psoralen (27 mg·kg^-1^), clopidogrel (7.5 mg·kg^-1^), and the same volume of 0.5% carboxy methyl cellulose. After 7 days, all rats were injected with 2.4 mg·kg^-1^ ciprofol. Compared with the control group, the AUC and MRT values of ciprofol in the psoralen and clopidogrel groups were significantly greater, whereas the CL values were significantly lower. In addition, the durations of loss of righting reflex (LORR) in the psoralen and clopidogrel groups were 16.1% and 23.0% longer than that in the control group, respectively. In conclusion, psoralen and clopidogrel inhibit ciprofol metabolism to different degrees and prolong the duration of LORR in rats.

## 1 Introduction

**Fig 1:**
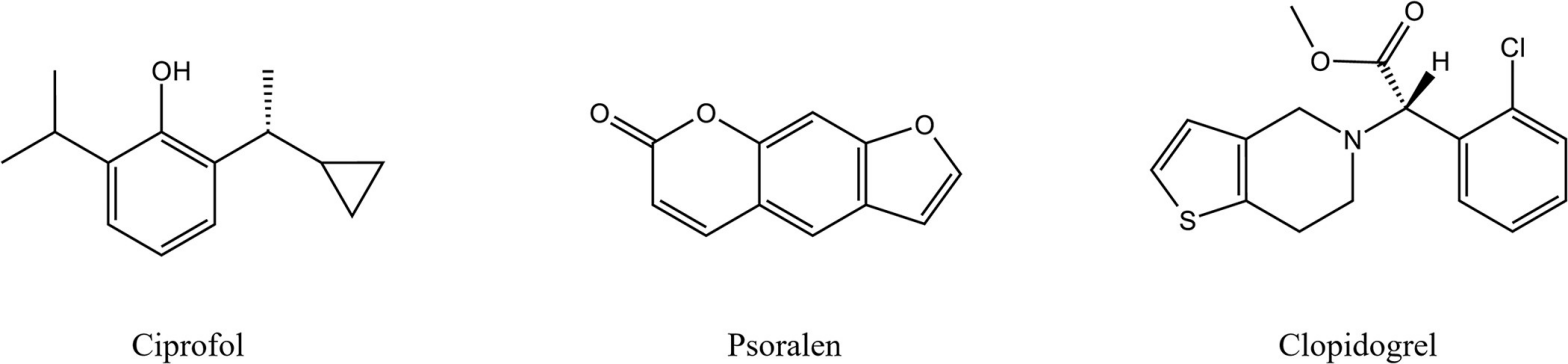
Chemical structures of ciprofol, psoralen and clopidogrel.

Ciprofol (HSK3486) is a novel 2,6-disubstituted phenol derivative short-acting intravenous anaesthetic [1], that has been approved for sedation and anaesthesia during nontracheal intubation, induction and maintenance of general anaesthesia, and sedation with mechanical ventilation during intensive care in China. Phase III trials of ciprofol for induction indications of general anaesthesia have been performed in the United States [2, 3]. Ciprofol is generated upon the addition of a cyclopropyl group to the side chain of propofol (Fig 1). This key structure not only reduces the lipophilicity of the parent structure by increasing the spatial effect but also breaks the symmetry of the original structure and forms a chiral centre, making the drug stereoselective with greater receptor affinity compared with propofol [1, 4]. The results of several clinical trials have shown that ciprofol can achieve the same anaesthetic effect with less lipid infusion volume, a lower risk of respiratory depression and cardiovascular adverse events at a quarter of the dose of propofol. The incidence of pain on injection caused by ciprofol is only 1/10 that caused by propofol. Thus, ciprofol exhibits good clinical application prospects [5–7].

Ciprofol is mainly metabolized by cytochrome P450 2B6 (CYP2B6) and uridine diphosphate glucuronosyltransferase 1A9 (UGT1A9), which form glucuronic acid monooxide and glucuronic acid conjugates, respectively [8]. With the increase in clinical applications, the combination of ciprofol and other drugs is becoming commonplace. Hou et al. [9] predicted that ciprofol did not cause drug‒drug interactions (DDIs) with the typical substrates of CYP1A2, CYP2B6, and CYP2C19 using in vitro-in vivo extrapolation (IVIVE) and physiologically based pharmacokinetic (PBPK) simulations. Clinical trials (NCT03758469, NCT04145583, and NCT05181007) have confirmed that rifampicin (a strong CYP2B6/UGT1A9 inducer), voriconazole (a weak CYP2B6 inhibitor), and mefenamic acid (a strong UGT1A9 inhibitor) have no clinical effect on ciprofol metabolism [10]. However, no reports have clarified whether CYP2B6 inactivators can affect ciprofol metabolism.

Psoralen and clopidogrel are moderate and efficient CYP2B6 inactivators, respectively, with irreversible inhibitory effects on CYP2B6 enzymes [11–13]. Psoralen (Fig 1) is the principal bioactive component in the dried and ripe fruits of *Psoralea corylifolia* L., which has been clinically used to treat a variety of skin diseases, such as psoriasis and vitiligo, and has a variety of biological activities, such as promoting bone regeneration, antitumour, antiviral, and anti-inflammatory activities [14, 15]. Clopidogrel (Fig 1), a thienopyridine P2Y12 receptor antagonist, is used for the secondary prevention of atherothrombotic events by blocking ADP-dependent platelet activation and aggregation to prevent thrombosis [16]. Patients who use ciprofol for anaesthesia or sedation are likely to receive treatment with psoralen or clopidogrel simultaneously given the extensive clinical application of these two drugs. Therefore, this study aimed to investigate the effects of psoralen and clopidogrel on ciprofol metabolism both in liver microsomes and in rats as convincing evidence of the DDIs of CYP2B6 inactivators and ciprofol and to provide a reference for the safety and efficacy of combination therapy in clinical practice.

## 2 Materials and methods

### 2.1 Drugs and reagents

Ciprofol (purity: 99.9%, 11220202), ciprofol injection (20 mL:50 mg, 20220207) and D_6_-ciprofol (purity: 99.8%, cc-HSK23287-202005006-001) were obtained from Haisco Pharmaceutical Co., Ltd. (Sichuan, China). Psoralen (purity: 99.6%, 110739–201918) and clopidogrel hydrogen sulfate (purity: 99.8%, 100819–201906) were purchased from the National Institutes for Food and Drug Control (Beijing, China). SD rat liver microsomes (20 mg·mL^-1^), male human liver microsomes (20 mg·mL^-1^), NADPH generation system (A, B) and phosphate-buffered saline (PBS) were produced by Wuhan PrimeTox Bio-pharma Technology Co. Ltd.

### 2.2 Animals

Healthy male Sprague‒Dawley rats (230 ± 20 g, licence number: SCXK (Beijing) 2019–0008) were purchased from Beijing Huafukang Biotechnology Co., Ltd. and housed under the following conditions: humidity of 50% ± 10%, a temperature of 25 ± 2°C, and a 12-h light/dark cycle. All rats had free access to food and water. The animal experiments were approved by the Research Ethics Committee of the Second Hospital of Hebei Medical University (Approval Letter No. 2023-AE285, date of approval: Aug 31, 2023). The experimental animals were euthanized through cervical dislocation following ciprofol anaesthesia (2.4 mg·kg^-1^). The operation was accurate and efficient, and the rats were continuously observed for abnormal reactions during the process to alleviate suffering.

### 2.3 Operating conditions for high-performance liquid chromatography-tandem mass spectrometry (HPLC-MS/MS)

The concentrations of analytes were determined using a HPLC-MS/MS system that consisted of high-performance liquid chromatography (Shimadzu, Japan, LC-20AD) and a triple quadrupole mass spectrometer (AB SCIEX, API 4000^+^, USA). The following liquid phase conditions were employed. A Symmetry C_18_ column (4.6 mm × 150 mm, 3.5 μm) was selected, and the column temperature was 40°C. The mobile phase consisted of acetonitrile (B) and 0.01% ammonia water (containing 5 mmol ammonium acetate) (A), with gradient elution at a flow rate of 0.7 mL·min^-1^. The gradient elution procedure was as follows: 0~1 min, 75%→99% B; 1~5.5 min, 99% B; and 5.5–6.5 min, 99%→75% B. The temperature of the automatic sampler was 4°C, and the sample volume was 10 μL. The internal standard (IS) was D_6_-ciprofol.

An electrospray power supply and negative ionization mode were used for multi-reaction monitoring. The specific mass spectrometry conditions were as follows: collision gas, 9 psi; curtain gas, 30 psi; ion spray voltage, -4500 V; temperature, 500°C; ion source gas 1, 50 psi; and ion source gas 2, 60 psi. The collision energies of ciprofol and D_6_-ciprofol were -28 V and -30 V, respectively, and the declustering potentials were maintained at -85 V and -82 V, respectively. The ion transitions were m/z 203.2→175.1 for ciprofol and m/z 209.3→181.3 for D_6_-ciprofol.

### 2.4 Establishment of the incubation system and sample processing method

#### 2.4.1 Liver microsomal incubation system

The volume of the incubation system was 200 μL and included 2 μL of substrate (ciprofol), 8 μL of rat liver microsomes (RLMs) or 6 μL of human liver microsomes (HLMs), 12 μL of NADPH generation system of solution (solution A 10 μL, solution B 2 μL), and the remaining volume was PBS. The incubation was performed in a water bath at a constant temperature of 37°C, and each sample was incubated three times in parallel.

#### 2.4.2 Processing of incubation system

The incubation system was removed from the water bath, and the reaction was terminated immediately by adding 800 μL of reaction termination solution (acetonitrile solution containing 5 μg·mL^-1^ D_6_-ciprofol at 4°C). The mixture was vortexed for 2 min and centrifuged for 15 min, after which the supernatant was transferred to an autosampler vial for HPLC-MS/MS detection.

#### 2.4.3 Processing of rat plasma samples

5 μL of IS solution (2.5 μg·mL^-1^ D_6_-ciprofol) was added to 100 μL of plasma and mixed. Then, 300 μL of acetonitrile was added. The mixture was vortexed for 2 min and centrifuged for 10 min, and the supernatant was transferred to an autosampler vial for HPLC-MS/MS detection.

### 2.5 Method validation

#### 2.5.1 Specificity

**I: A** Liver microsome incubation system without adding ciprofol. **B** Incubation system sample spiked with ciprofol. **C** Incubation system sample spiked with IS. Then, 800 μL of acetonitrile was added to the above samples, which were subsequently vortexed for 2 min and centrifuged for 15 min. The supernatant was analysed. **D** Samples from the actual incubation system were treated as described in Section 2.4.2 and then injected for assessment.

**II: A** Blank rat plasma. **B** Blank rat plasma spiked with ciprofol. **C** Blank rat plasma spiked with IS. Then, 300 μL of acetonitrile was added to the above samples, which were subsequently vortexed for 2 min and centrifuged for 15 min. Then, the supernatant was detected. **D** After the administration of 2.4 mg·kg^-1^ ciprofol, the plasma samples from the rats were treated as described in Section 2.4.3 and injected for assessment.

#### 2.5.2 Standard curves and lower limit of quantification (LLOQ)

Liver microsomes were inactivated in a water bath at 100°C for 30 min and diluted with PBS to obtain an inactive incubation system. Then, an appropriate amount of ciprofol standard was dissolved into ciprofol solution with acetonitrile and added to the inactive incubation system to prepare ciprofol liver microsomal sample solutions at concentrations of 1.0, 2.5, 5.0, 10, 25, 50 and 100 μg·mL^-1^. The sample solutions were processed and assessed as described in Section 2.4.2. Additionally, an appropriate amount of ciprofol solution was added to the blank plasma of rats to prepare simulated plasma samples at concentrations of 15, 30, 60, 120, 250, 500, 1000 and 2000 ng·mL^-1^. These samples were processed and analysed as described in Section 2.4.3. The ciprofol concentration (*x*) was reported as the abscissa, and the ratio of the peak area (*y*) of ciprofol to the IS served as the ordinate. The concentration of ciprofol was set as the LLOQ when the signal-to-noise ratio (*S*/*N*) was ≥10.

#### 2.5.3 Precision, accuracy, recovery rate and matrix effect

The ciprofol liver microsome quality control (QC) samples were prepared at 2.5, 10 and 75 μg·mL^-1^ from the inactive incubation system and were treated and evaluated as described in Section 2.4.2. Ciprofol simulated plasma QC samples were prepared at concentrations of 30, 250 and 1600 ng·mL^-1^ and processed and assayed as described in Section 2.4.3. Five samples of each concentration were processed and measured on one day, and the intra-batch precision and accuracy were calculated. The samples were processed and measured for 3 consecutive days, and the inter-batch precision and accuracy were calculated. The precision was expressed as the relative standard deviation (RSD), and the accuracy was expressed as the relative error (RE). The absolute recovery was calculated according to the ratio of the peak area of ciprofol in the QC sample to the peak area of the same amount of solution of the ciprofol standard injected directly.

The blank incubation system and rat blank plasma were processed as described in Sections 2.4.2 and 2.4.3 (without IS), respectively. The supernatant was collected to obtain the blank matrix solutions. Low, medium and high concentrations of the QC solution and IS solutions were added to the blank matrix solutions at the same concentration as the liver microsome QC samples and simulated plasma QC samples of ciprofol, respectively. This procedure was repeated after replacing the blank substrate with 50% acetonitrile. The matrix effect was calculated based on the ratio of the peak area of ciprofol and the IS in the blank matrix to the peak area of the solution of the equivalent ciprofol standard.

#### 2.5.4 Stability

To investigate the stability of ciprofol under a variety of conditions, three groups of liver microsome QC sample solutions were prepared, and one group was immediately processed and analysed as described in Section 2.4.2. Another group was stored at room temperature for 8 hours before treatment and assessment. The other group was treated and placed in the autosampler (4°C) for 24 h and subsequently analysed. In addition, simulated plasma QC samples were prepared in five groups: one group was processed and measured immediately as described in Section 2.4.3, and another group was placed in the autosampler for 24 h after treatment. The other three groups were placed at room temperature for 8 h, stored at -40°C for 30 d, frozen and thawed three times, respectively, and then processed and detected as described in Section 2.4.3. All the above samples were tested in parallel in five batches.

### 2.6 The effects of psoralen and clopidogrel on ciprofol metabolism in liver microsomes

#### 2.6.1 Incubation conditions

The concentrations of ciprofol and liver microsomal proteins in the RLM incubation system were set at 25 μg·mL^-1^ and 0.8 mg·mL^-1^, respectively, whereas those in the HLM incubation system were set at 40 μg·mL^-1^ and 0.6 mg·mL^-1^, respectively. Samples were obtained and assayed after 0, 10, 20, 30, 40, 60, 80, 100 and 120 min of incubation, and the optimum incubation time was determined according to the decreasing trend in ciprofol concentration.

The ciprofol concentrations in the RLM and HLM incubation systems were set at 25 and 40 μg·mL^-1^, respectively. The liver microsomal protein concentrations were 0, 0.1, 0.2, 0.4, 0.6, 0.8 and 1.0 mg·mL^-1^. The optimal protein concentration in the incubation system was determined based on the reduction of ciprofol (ΔC) after 40 min of incubation in RLMs and 60 min in HLMs.

#### 2.6.2 Kinetic parameters of ciprofol metabolism

The liver microsomal protein concentrations in the RLM incubation system and the HLM incubation system were 0.8 mg·mL^-1^ and 0.6 mg·mL^-1^, and the cells were incubated for 40 min and 60 min, respectively. The ciprofol concentrations in the incubation system were set at 4, 8, 16, 32, 48 and 80 μg·mL^-1^, and the kinetic parameters of ciprofol metabolism were investigated based on ΔC values at different initial ciprofol concentrations (C_0_).

#### 2.6.3 The effects of psoralen and clopidogrel on ciprofol metabolism in liver microsomes

The incubation system was prepared according to the optimal incubation conditions, and different concentrations of psoralen or clopidogrel solution were added to the blank incubation system at concentrations of 0, 5, 12.5, 25, 50, 75, 100, 150, and 200 μmol·L^-1^ and 0, 0.2, 0.5, 1, 2, 5, 10, 25, and 50 μmol·L^-1^, respectively. The incubation system was preincubated in a water bath (37°C) for 10 min, and ciprofol was added to the system and subsequently incubated subsequently. The effects of psoralen and clopidogrel on the metabolism of ciprofol were investigated based on ΔC.

### 2.7 The effects of psoralen and clopidogrel on the pharmacokinetics and pharmacodynamics of ciprofol in rats

Twenty-four SD rats were randomly and equally divided into three groups: the control group, psoralen group and clopidogrel group. The administration scheme was as follows: the control group received 0.5% carboxy methyl cellulose by gavage (1 mL·200 g^-1^), the psoralen group received 27 mg·kg^-1^ psoralen by gavage, and the clopidogrel group was gavaged with 7.5 mg·kg^-1^ clopidogrel. The dosage was calculated according to the results of previous studies, and the human dosage was translated into the equivalent rat dosage according to the Chinese pharmacopoeia and drug instructions [17–21]. All rats were treated once daily for seven days. Each rat was injected with 2.4 mg·kg^-1^ ciprofol via the tail vein 12 h after the end of dosing on Day 7. Rats that received ciprofol injection were placed on a flat and soft insulation pad. Timekeeping began when the rats were in the supine position and lost the righting reflex and ended when the rats could turn to the prone position successfully. The duration of loss of righting reflex (LORR) in each rat was measured [22]. During this period, approximately 300 μL of blood was collected from the inner canthus at 2, 4, 8, 12, 16, 20, 30, 45, and 60 min after ciprofol administration, and the plasma was separated. The plasma was processed and measured as described in Section 2.4.3, and *DAS* 2.0 software and a noncompartmental model were used to analyse the pharmacokinetic parameters.

### 2.8 Data statistics

The substrate elimination method was employed in this study, and *GraphPad Prism* 8.0.2 software was used for statistical processing, including determination of the kinetic parameters of ciprofol metabolism and the *IC*_50_ values of psoralen and clopidogrel for ciprofol metabolism. The maximum velocity (*V*_max_), Michaelis constant (*K*_m_) and intrinsic clearance (*CL*_int_) were calculated using the Michaelmas equation:

$$V=\frac{V_{max}\times [S]}{K_{m}+[S]}$$

$${CL}_{int}=V_{max}/K_{m}$$

*V* and [*S*] represent the reaction rate and concentration of the substrate in the formula, respectively.

*IBM SPSS Statistics* 27 software was used to analyse the main pharmacokinetic parameters, and *GraphPad Prism* 8.0.2 software was used for graphical visualization. One-way ANOVA was used, and multiple comparisons were performed using the least significant difference (LSD)-T test, for which *P* < 0.05 indicated a significant difference.

## 3 Results

### 3.1 Method validation

**Fig 2:**
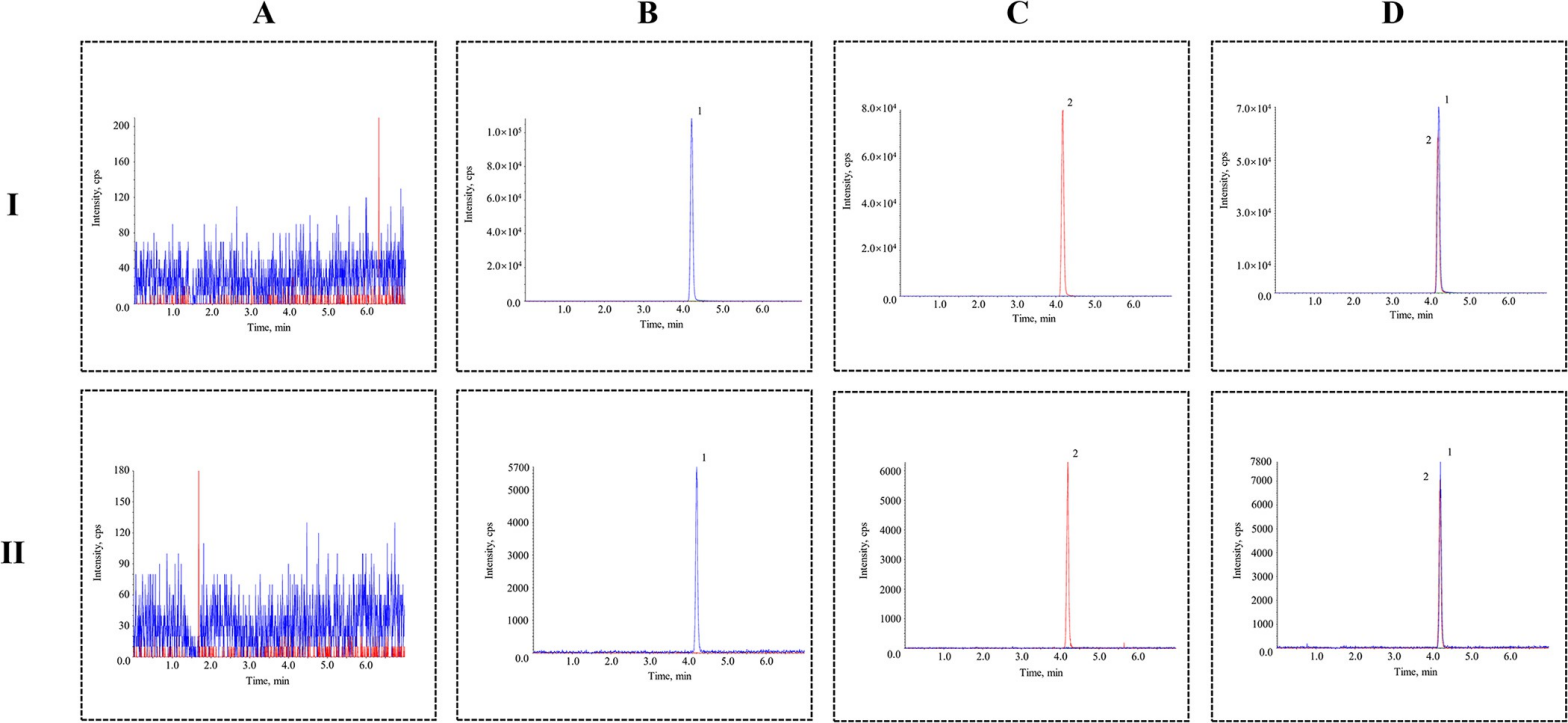
HPLC-MS/MS spectra of liver microsome incubation system samples (I) and rat plasma samples (II). (**A**) Blank substrate samples. (**B**) Blank substrate samples spiked with ciprofol. (**C**) Blank substrate samples spiked with IS. (**D**) Samples actually measured; 1: ciprofol (blue), 2: IS (red).

Methods for the determination of ciprofol in liver microsomal incubation system and rat plasma were investigated. The results showed that the retention times of both ciprofol and the IS were approximately 4.21 min. Neither endogenous substances in the liver microsomes nor rat plasma interfered with the analytes (Fig 2).

The ciprofol concentrations in the liver microsome incubation system and rat plasma were in the linear range of 1–100 μg·mL^-1^ and 15–2000 ng·mL^-1^, and the LLOQs were 1 μg·mL^-1^ and 15 ng·mL^-1^, respectively. The standard curve equations were as follows:

$$Liver\;microsomes:\;y=1.03\times 10^{-1}x-6.51\times 10^{-2};\;r2=0.999$$ 1

$$Rat\;plasma:\;y=1.907\times 10^{-3}x+5.05\times 10^{-4};\;r2=0.999$$ 2

The accuracies of ciprofol detection in liver microsomes and rat plasma were 96.6–104.2% and 96.1–105.9%, respectively. The RSD values for intra-batch and inter-batch precision were less than 6%. The absolute recoveries were all greater than 90%, with RSDs less than 8%. The matrix effects of ciprofol were within the range of ± 8% after correction by the IS, and the RSDs were all less than 10% (Table 1). In addition, the QC samples exhibited satisfactory stability under various conditions (Table 2).

**Table 1:** Precision, accuracy, absolute recovery and matrix effect of ciprofol in liver microsome incubation system and rat plasma.

Matrix solution	QC concentration	Intra-batch			Inter-batch			Absolute Recovery		Matrix Effect	
		Mean ± SD	RSD (%)	RE (%)	Mean ± SD	RSD (%)	RE (%)	Mean ± SD (%)	RSD (%)	Mean ± SD (%)	RSD (%)
**Liver microsomes**	2.5 μg·mL^-1^	2.61 ± 0.15	5.87	104.2	2.53 ± 0.07	2.60	101.3	92.42 ± 7.25	7.84	92.07 ± 6.32	6.87
	10 μg·mL^-1^	10.35 ± 0.37	3.54	103.5	10.17 ± 0.23	2.23	101.7	90.59 ± 5.16	5.69	103.6 ± 6.1	5.88
	75 μg·mL^-1^	72.46 ± 3.48	4.80	96.6	76.02 ± 2.84	3.73	101.4	91.09 ± 3.12	6.79	94.97 ± 9.32	9.81
**Rat plasma**	30 ng·mL^-1^	31.88 ± 1.68	5.26	102.0	33.10 ± 1.09	3.29	105.9	93.18 ± 5.93	6.37	94.85 ± 6.88	7.25
	250 ng·mL^-1^	240.36 ± 6.56	2.73	96.1	247.13 ± 10.46	4.23	98.9	98.66 ± 5.74	5.82	100.6 ± 7.7	7.68
	1600 ng·mL^-1^	1594.18 ± 55.87	3.50	99.6	1619.78 ± 38.12	2.35	101.2	96.84 ± 4.03	4.17	96.07 ± 6.35	6.60

**Table 2:** Stability of ciprofol in liver microsome incubation system and in plasma under various storage conditions (n = 5).

Matrix solution	QC concentration	Room Temperature ^a^		Autosampler ^b^		Freeze–Thaw ^c^		Long-Term Storage ^d^	
		Mean ± SD	RSD (%)	Mean ± SD	RSD (%)	Mean ± SD	RSD (%)	Mean ± SD	RSD (%)
**Liver microsomes**	2.5 μg·mL^-1^	2.53 ± 0.19	7.41	2.54 ± 0.11	4.41	*NA*		*NA*	
	10 μg·mL^-1^	10.14 ± 0.62	6.15	9.82 ± 0.44	4.48				
	75 μg·mL^-1^	70.43 ± 3.12	4.43	72.07 ± 4.83	6.71				
**Rat plasma**	30 ng·mL^-1^	30.50 ± 2.27	7.45	32.80 ± 1.93	5.89	33.49 ± 2.03	6.07	32.70 ± 2.85	8.72
	250 ng·mL^-1^	227.31 ± 5.60	2.46	250.78 ± 12.31	4.91	245.21 ± 3.43	1.40	242.73 ± 9.08	3.74
	1600 ng·mL^-1^	1464.24 ± 98.79	6.75	1576.27 ± 41.67	2.64	1604.92 ± 96.88	6.04	1597.51 ± 83.93	5.25

Notes: ^a^ room temperature for 8 h

^b^ autosampler for 24 h after treatment

^c^ three freeze–thaw cycles

^d^ −40°C for 30 days.

### 3.2 The effects of psoralen and clopidogrel on ciprofol metabolism in liver microsomes

#### 3.2.1 Kinetic parameters of ciprofol metabolism

**Fig 3:**
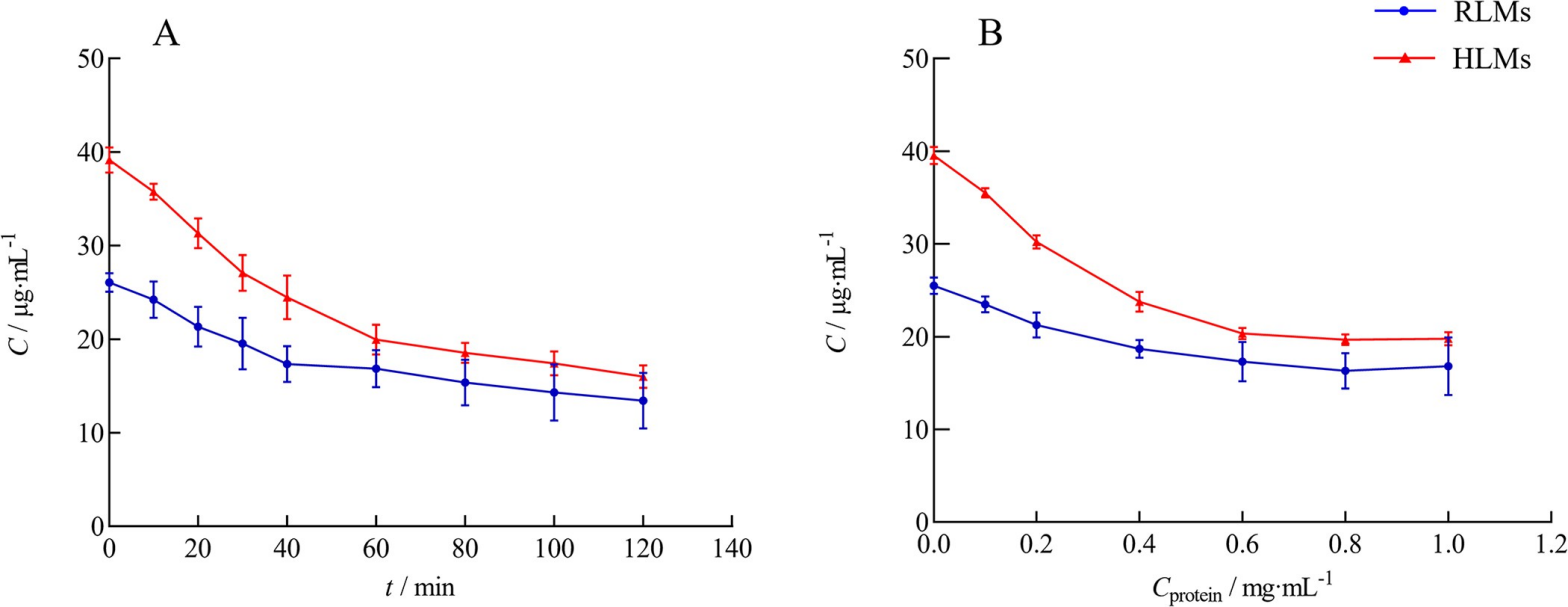
Effects of incubation time and protein concentration on ciprofol metabolism in the liver microsome incubation system (n = 3). (A) Various incubation times. (B) Various protein concentrations.

Based on the trends of the residual concentration of ciprofol in the incubation system under different incubation times and different liver microsomal protein concentrations (Fig 3), the optimal incubation time and liver microsomal protein concentration in the RLMs were 40 min and 0.8 mg·mL^-1^, respectively. For the HLMs, the optimal incubation time was 60 min, and the concentration of liver microsomal protein was 0.6 mg·mL^-1^.

**Fig 4:**
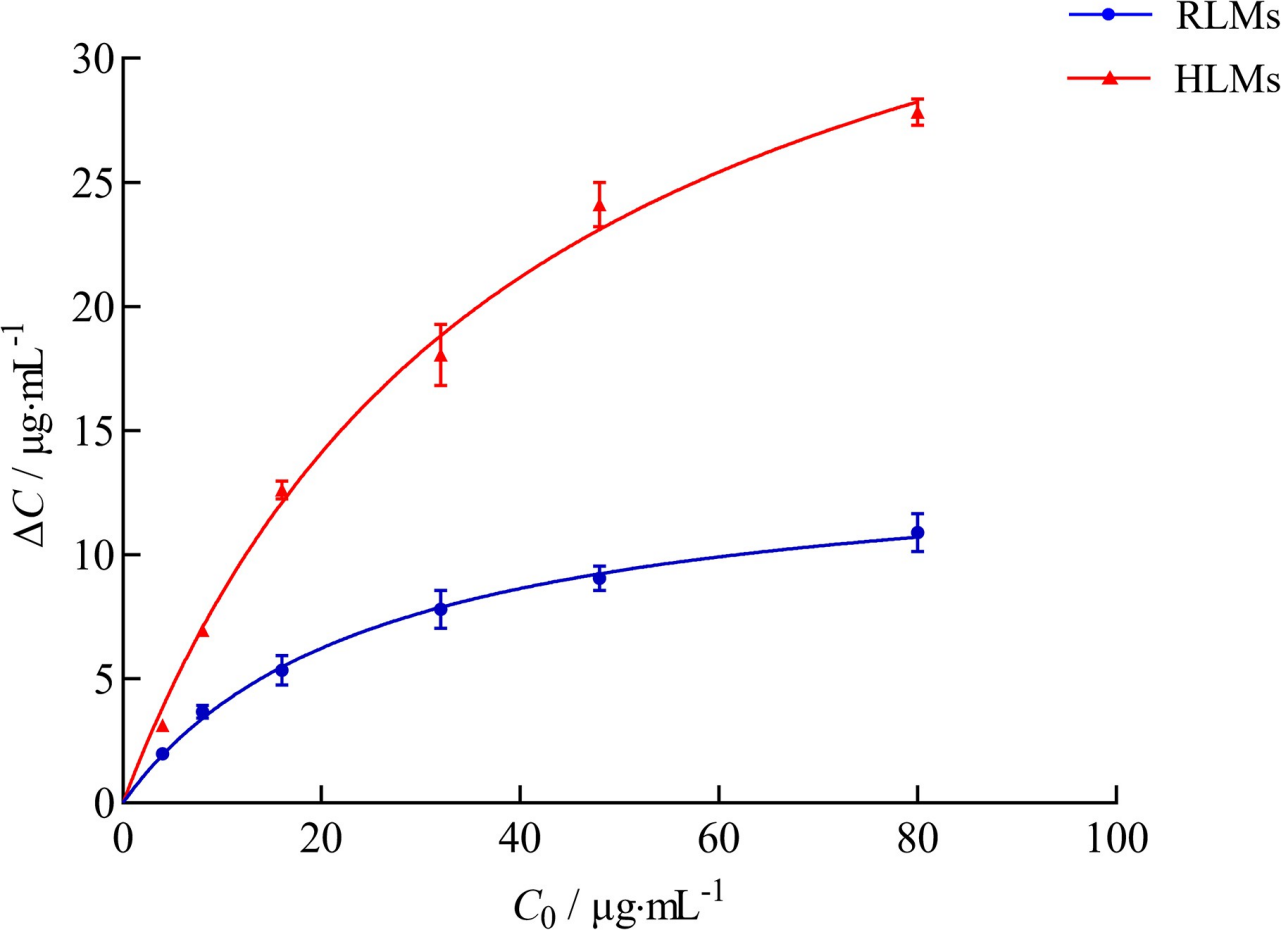
Enzyme kinetic curve of ciprofol in RLMs and HLMs (n = 3).

As shown in Fig 4, ΔC increased significantly with increasing C_0_ initially, after which the trend slowed. The results indicated that ciprofol metabolism in RLMs and HLMs was best described by Michaelis‒Menten kinetics, with *K*_m_ values of 25.11 μg·mL^-1^ and 40.07 μg·mL^-1^ and *V*_max_ values of 14.06 ng·min^-1^·mg protein^-1^ and 42.39 ng·min^-1^·mg protein^-1^, respectively. The *CL*_int_ values of ciprofol in the RLMs and HLMs were 0.56 and 1.06 mL·min^-1^·mg protein^-1^, respectively. The HLMs exhibited greater ciprofol metabolic activity compared with RLMs.

#### 3.2.2 The effects of psoralen and clopidogrel on ciprofol metabolism in liver microsomes

**Fig 5:**
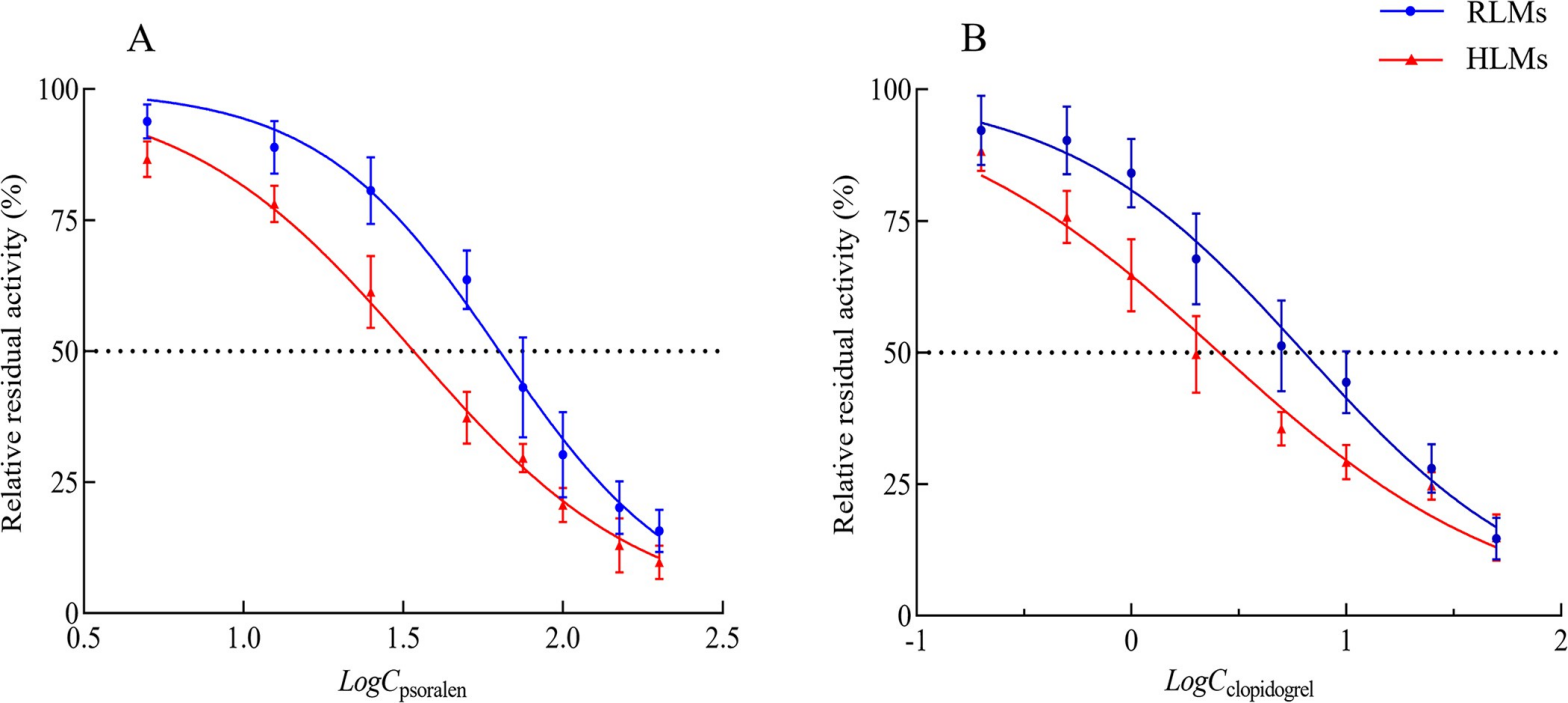
Effects of psoralen and clopidogrel on ciprofol metabolism in liver microsome incubation system (n = 3). (**A**) Effects of psoralen on metabolic activities. (**B**) Effect of clopidogrel on metabolic activities.

Psoralen and clopidogrel decreased ciprofol metabolism in the liver microsomal incubation system in a concentration-dependent manner, and the inhibitory effects in HLMs were stronger than those in RLMs (Fig 5A and 5B). Specifically, the *IC*_50_ values of psoralen were 63.31 μmol·L^-1^ and 34.05 μmol·L^-1^ for ciprofol in RLMs and HLMs, respectively, showing mild inhibitory effects. However, the *IC*_50_ values of clopidogrel were 6.380 μmol·L^-1^ and 2.565 μmol·L^-1^ for ciprofol in RLMs and HLMs, respectively, indicating moderate inhibitory effects [23].

### 3.3 The effects of psoralen and clopidogrel on the pharmacokinetics and pharmacodynamics of ciprofol in rats

#### 3.3.1 Pharmacokinetics

**Fig 6:**
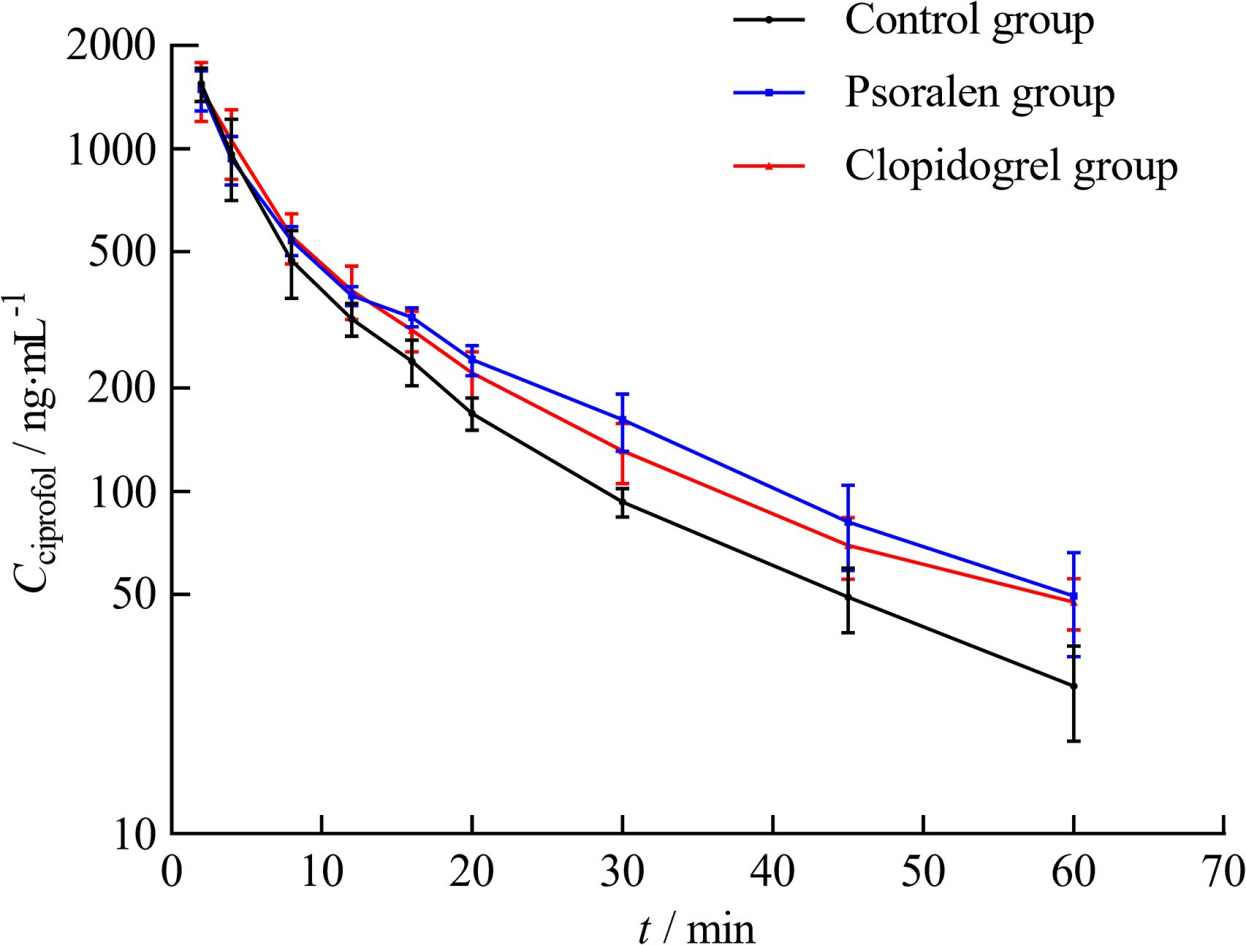
The mean plasma concentration–time curves of ciprofol in different groups (n = 8). Ciprofol (2.4 mg·kg^-1^, i.v.) was administered in the control group. Ciprofol (2.4 mg·kg^-1^, i.v.) was co-administered with psoralen (27 mg·kg^-1^, oral) in the psoralen group. Ciprofol (2.4 mg·kg^-1^, i.v.) was co-administered with clopidogrel (7.5 mg·kg^-1^, oral) in the clopidogrel group.

The mean plasma concentration–time curves of ciprofol in the control, psoralen, and clopidogrel groups are shown in Fig 6. The ciprofol concentration decreased rapidly, with a more obvious decreasing trend observed in the control group.

As shown in Table 3, compared with those in the control group, the AUC_0-t_ and AUC_0-∞_ in the psoralen group increased by 15.3% and 17.7% (*P* < 0.05), respectively. In addition, the MRT_0-t_ and MRT_0-∞_ increased by 26.1% (*P* < 0.01) and 31.6% (*P* < 0.05), respectively, and the CL decreased by 15.8% (*P* < 0.05). The AUC_0-∞_ of the clopidogrel group increased by 18.8% compared with that of the control group (*P* < 0.05). MRT_0-t_ and MRT_0-∞_ increased by 16.7% (*P* < 0.05) and 45.1% (*P* < 0.01), respectively, whereas CL decreased by 15.8% (*P* < 0.05). However, no significant differences in the C_max_, Vd or t_1/2_ of ciprofol were noted among the three groups. The pharmacokinetic parameters of ciprofol in rats in the two experimental groups were also not significantly different.

**Table 3:** Pharmacokinetic parameters of ciprofol in rats (n = 8).

Pharmacokinetic parameters	Statistical data of each group (Mean ± SD)		
	Control group	Psoralen group	Clopidogrel group
AUC_0-t_ (μg·h·L^-1^)	259.74 ± 35.07	299.49 ± 17.56*	295.85 ± 40.78
AUC_0-∞_ (μg·h·L^-1^)	267.70 ± 35.53	315.09 ± 23.45*	318.03 ± 44.96*
MRT_0-t_ (h)	0.18 ± 0.01	0.23 ± 0.02**	0.21 ± 0.02**
MRT_0-∞_ (h)	0.22 ± 0.04	0.28 ± 0.05*	0.31 ± 0.11**
t_1/2_ (h)	0.23 ± 0.05	0.26 ± 0.05	0.35 ± 0.23
CL (L·h^-1^·kg^-1^)	9.12 ± 1.20	7.68 ± 0.54*	7.68 ± 1.20*
Vd (L·kg^-1^)	3.02 ± 0.72	2.80 ± 0.46	3.71 ± 2.03
C_max_ (ng·mL^-1^)	1546.59 ± 172.72	1489.71 ± 200.05	1494.12 ± 291.67

* *P* < 0.05

** *P* < 0.01; compared with the control group, indicating a statistically significant difference. AUC: area under the curve; MRT: mean residence time; t_1/2_: elimination half-life; CL: plasma clearance; Vd: volume of distribution; C_max_: peak concentration.

#### 3.3.2 Pharmacodynamics

**Fig 7:**
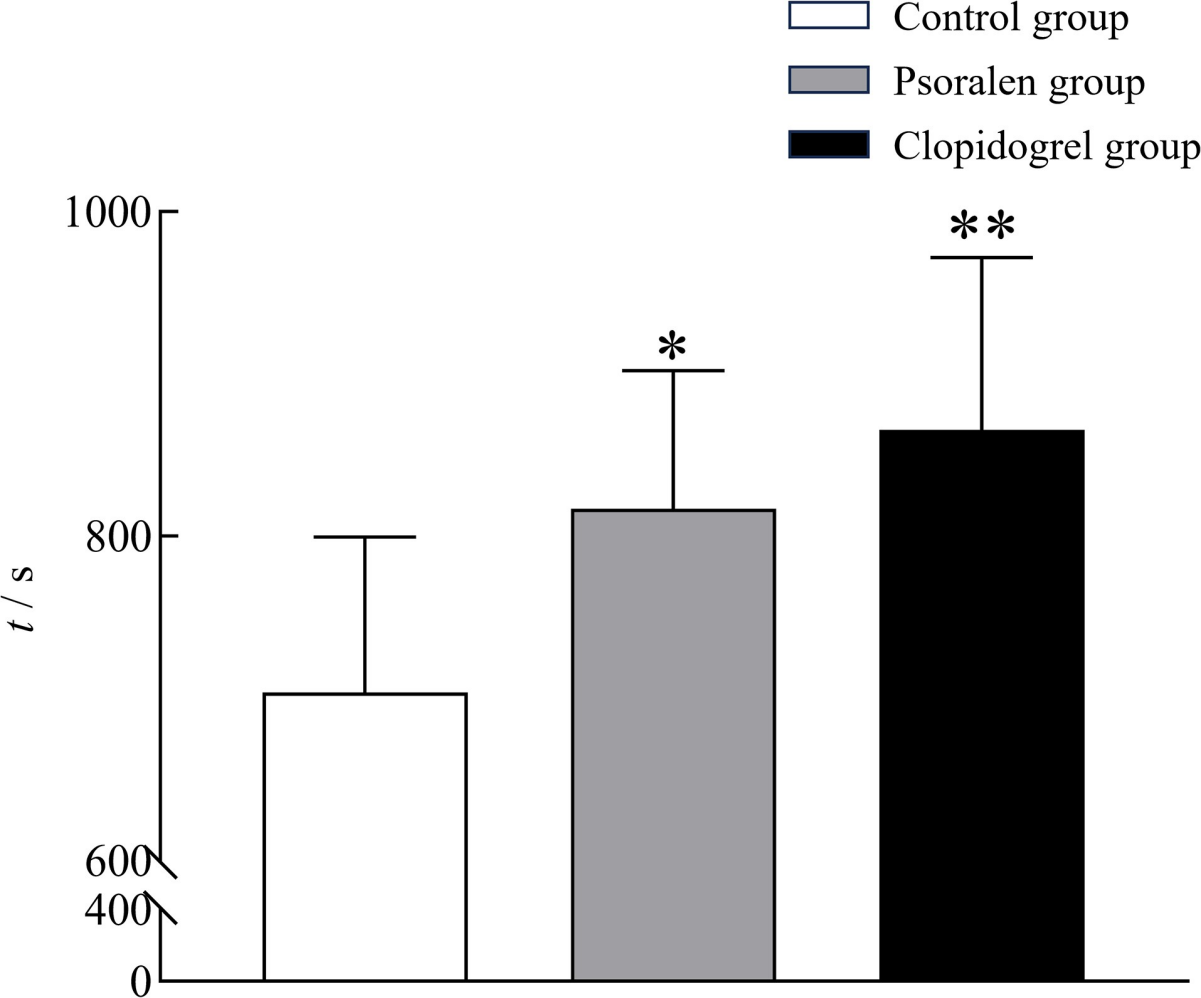
The durations of LORR in each group of rats (n = 8). * *P* < 0.05; ** *P* < 0.01, compared with the control group.

All rats lost their righting reflex rapidly after ciprofol injection through the caudal vein. The duration of LORR in the control group was 703.63 ± 95.94 s, whereas the durations of LORR in the psoralen group and the clopidogrel group were significantly longer (817.00 ± 85.02 s, *P* < 0.05; 865.63 ± 106.20 s, *P* < 0.01; respectively). No significant difference was noted between the two experimental groups (*P* = 0.323) (Fig 7).

## 4 Discussion

Ciprofol is a novel short-acting intravenous anaesthetic [24]. Given its advantages in terms of safety, ciprofol has already played an important role in clinical sedation and anaesthesia in China, and there is a trend toward further expansion [25, 26]. Although studies on ciprofol are becoming more extensive and in depth, no studies on methods for determining ciprofol concentrations have been reported to date. Therefore, a HPLC-MS/MS method that can be used to determine the ciprofol concentration in liver microsomal incubation system and rat plasma was developed in this study. Given that ciprofol is a weakly acidic compound, 0.01% ammonia was added to the aqueous phase (A) to enhance the response signal by providing a weakly alkaline environment (pH ≈ 8.1). A sufficient amount of ammonium ions were supplied upon the addition of ammonium acetate to the aqueous phase, resulting in less tailing of the chromatographic peak and a 50% greater response signal. In addition, the loss of life of the chromatographic column can be avoided due to excessive alkali. Moreover, we used acetonitrile to replace methanol as the organic phase (B) because of the lower background noise and stronger elution ability, which can decrease the width of the peak of the tested substance and improve the peak shape. Signal acquisition of the analytes was performed in a negative ion and multi-reaction monitoring mode. The use of stable isotope-labelled IS maximized the elimination of ionization differences and reduced matrix effects. Ciprofol exhibits minimal polarity, and it is easily dissolved in methanol or acetonitrile. The protein precipitation method is easy to perform, and the supernatant is cleaner when acetonitrile is used as a precipitant than when methanol is used.

Although historically regarded as a minor cytochrome P450 enzyme in the human liver, accumulating evidence has demonstrated that CYP2B6 plays an important role in human drug metabolism. Many drugs with inhibitory effects on CYP2B6 activity may affect the metabolism of CYP2B6 substrates [27, 28]. Ji et al. [13] proposed that psoralen is a mechanism-based CYP2B6 inactivator that can moderately inhibit the activity of the recombinant human CYP2B6 enzyme. The inactivating effect is not produced by psoralen itself, rather its furan epoxy derivatives formed by epoxidation, or γ-ketonene formed by direct oxidation. In addition, the mechanism of inactivation was previously reported [29, 30]. In addition, previous studies have chosen the CYP2B6 probe drug bupropion as a metabolic substrate and demonstrated that clopidogrel strongly inhibits recombinant human CYP2B6 enzyme activity. The inactivation potency of the prototype clopidogrel drug is 11 times greater than that of its thiolactone metabolite [31, 32]. Mixed liver microsomes, which are more closely related to the true composition and actual concentration of each metabolic enzyme in the liver, were selected for this study, and it was concluded that psoralen and clopidogrel had mild and moderate inhibitory on the metabolism of ciprofol, respectively. This study revealed that the clearance of ciprofol in rats decreased after 7 days of intragastric administration of psoralen or clopidogrel, and the MRT was significantly longer than that of the control group, leading to increased exposure. In addition, the LORR of the rats in the psoralen and clopidogrel groups was prolonged by 16.1% and 23.0%, respectively, compared with that of the control group, suggesting that CYP2B6 inactivators could inhibit the metabolism of ciprofol to some extent in rats.

As the main component of various Chinese patent medicines [33–36], psoralen has a variety of biological activities and is widely used, especially in middle-aged and elderly people. Therefore, patients taking psoralen-containing medicines may be administered ciprofol during sedation/anaesthesia for examination or surgery. Similarly, clopidogrel, one of the most common antiplatelet drugs, may be used in combination with ciprofol in patients undergoing fiberoptic bronchoscopy, gastroscopy, colonoscopy, and mechanical ventilation. Of note, the administration of clopidogrel is usually suspended 3 to 7 days before surgery, depending on the type of surgery [37, 38], whereas a growing number of studies provide a different perspective. A multidisciplinary expert consensus on the perioperative management of antithrombotic drugs (2020) issued by China recommends that patients with high risks of thrombosis and low risks of bleeding, such as patients with ischaemic stroke or transient ischaemic attacks, should take clopidogrel consistently during the perioperative period [39]. A meta-analysis showed that continuation of antiplatelet therapy did not have a significant impact on bleeding complications in patients undergoing noncardiac surgery with important indications [40]. Nijjer et al. [41] conducted a meta-analysis of 34 studies and found that patients with acute coronary syndromes could undergo coronary artery bypass grafting without cessation of clopidogrel administration. In addition, several studies have demonstrated the safety of continuing to use clopidogrel during the perioperative period in patients undergoing gastrointestinal surgery, urethral resection of the prostate, and hip and knee arthroplasty [42–44]. Given these findings, we cannot eliminate the possibility of the combined administration of clopidogrel and ciprofol in patients undergoing urgent or elective surgery.

DDI studies on ciprofol have attracted increasing attention due to its growing application in China. Available evidence shows that administering ciprofol for anaesthesia induction may not necessitate additional precautions in patients taking concomitant medications metabolized by CYP2B6 enzymes, such as acetaminophen, S-mephenytoin and bupropion [9]. However, the results of our study suggest that CYP2B6 inactivators have the potential to inhibit the metabolism of ciprofol. This finding is a good complement to ciprofol-associated DDIs. Considering the substantial difference in metabolic environments in vivo and in vitro, as well as the species differences between humans and rats, this conclusion needs to be further validated by more extensive and in-depth clinical trials.

## 5 Conclusions

In this study, a HPLC-MS/MS method for assessing ciprofol concentration in liver microsomal incubation system and rat plasma was established. This method was successfully applied to DDI studies between ciprofol and psoralen or clopidogrel. The results indicated that psoralen and clopidogrel can inhibit ciprofol metabolism to different degrees and prolong the duration of LORR in rats. Considering the increasing clinical application of ciprofol, attention should be given to whether the combination of CYP2B6 inactivators with ciprofol could affect the recovery time and physiological indicators of patients under clinical sedation or anaesthesia and whether it would cause adverse reactions after surgery.

## Supporting information

S1 File (DOCX)

S1 Raw data (XLSX)
