# Supplementary material for: The effects of CYP2B6 inactivators on the metabolism of ciprofol
Source: PLoS One. 2024 Jul 29;19(7):e0307995. doi: 10.1371/journal.pone.0307995 (PMC11285948; doi:10.1371/journal.pone.0307995)

**Log Information from Devices at Start of acquisition:**

**Shimadzu LC Method Properties**

Shimadzu LC system Equlibration time = 3.00 min

Shimadzu LC system Injection Volume = 10.00 ul

Shimadzu LC Method Parameters

Pumps

=====

Pump A Model: LC-20AD

Pump B Model: LC-20AD

Pumping Mode: Binary Flow

Total Flow: 0.7000 mL/min

Pump B Conc: 75.0 %

B Curve: 0

Pressure Range (Pump A/B): 0 - 2898 psi

Pump A Compressability: Off

Pump B Compressability: Off

Autosampler

===========

Model: SIL-20AC/HT

Use Autosampler: Yes

Rinsing Volume: 200 uL

Needle Stroke: 45 mm.

Rinsing Speed: 35 uL/sec

Sampling Speed: 15.0 uL/sec

Purge Time: 25.0 min

Rinse Dip Time: 5 sec

Rinse Mode: Before and after aspiration

Cooler Enabled: Yes

Cooler Temperature: 4 deg. C

Control Vial Needle Stroke: 52 mm


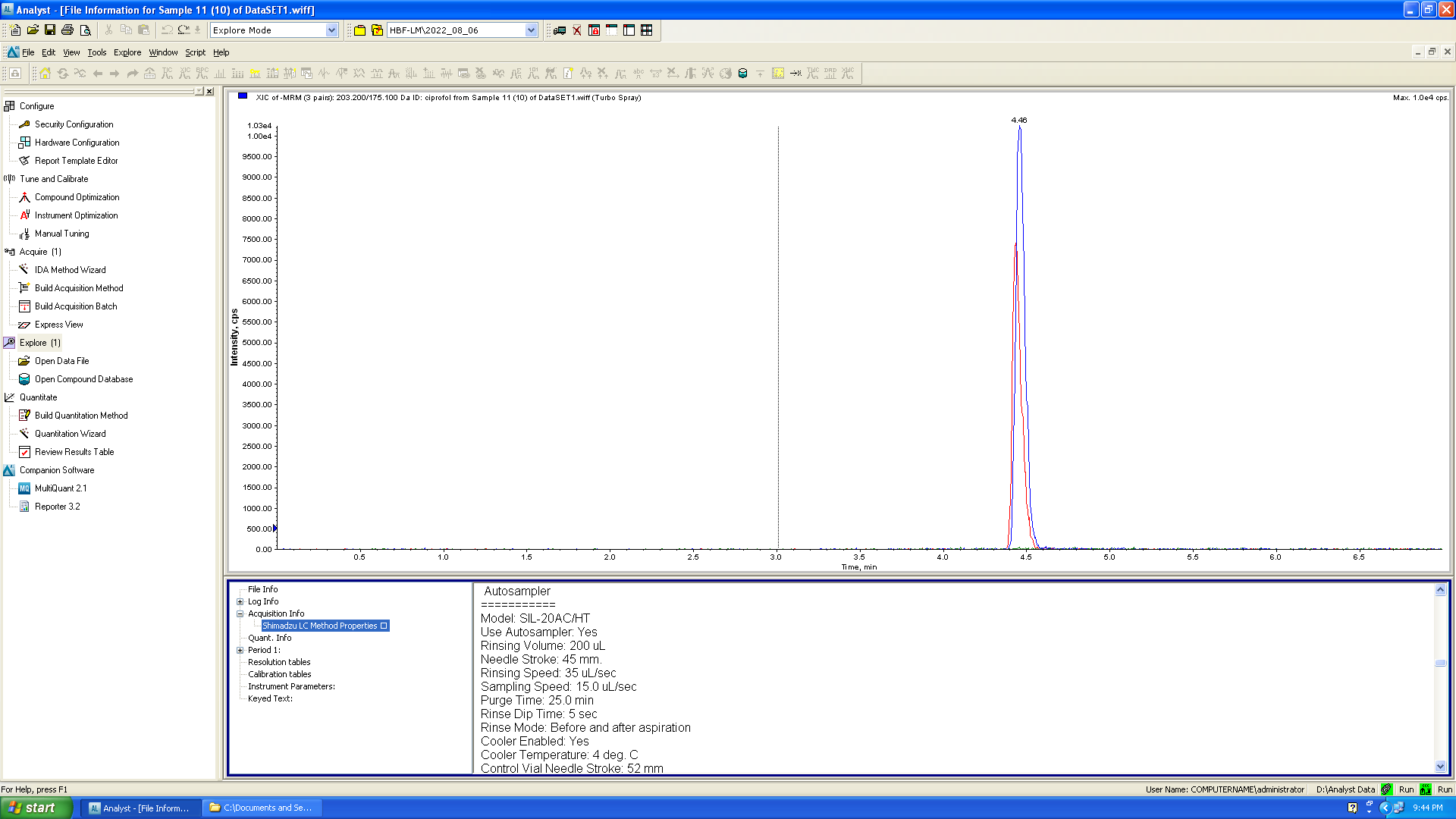


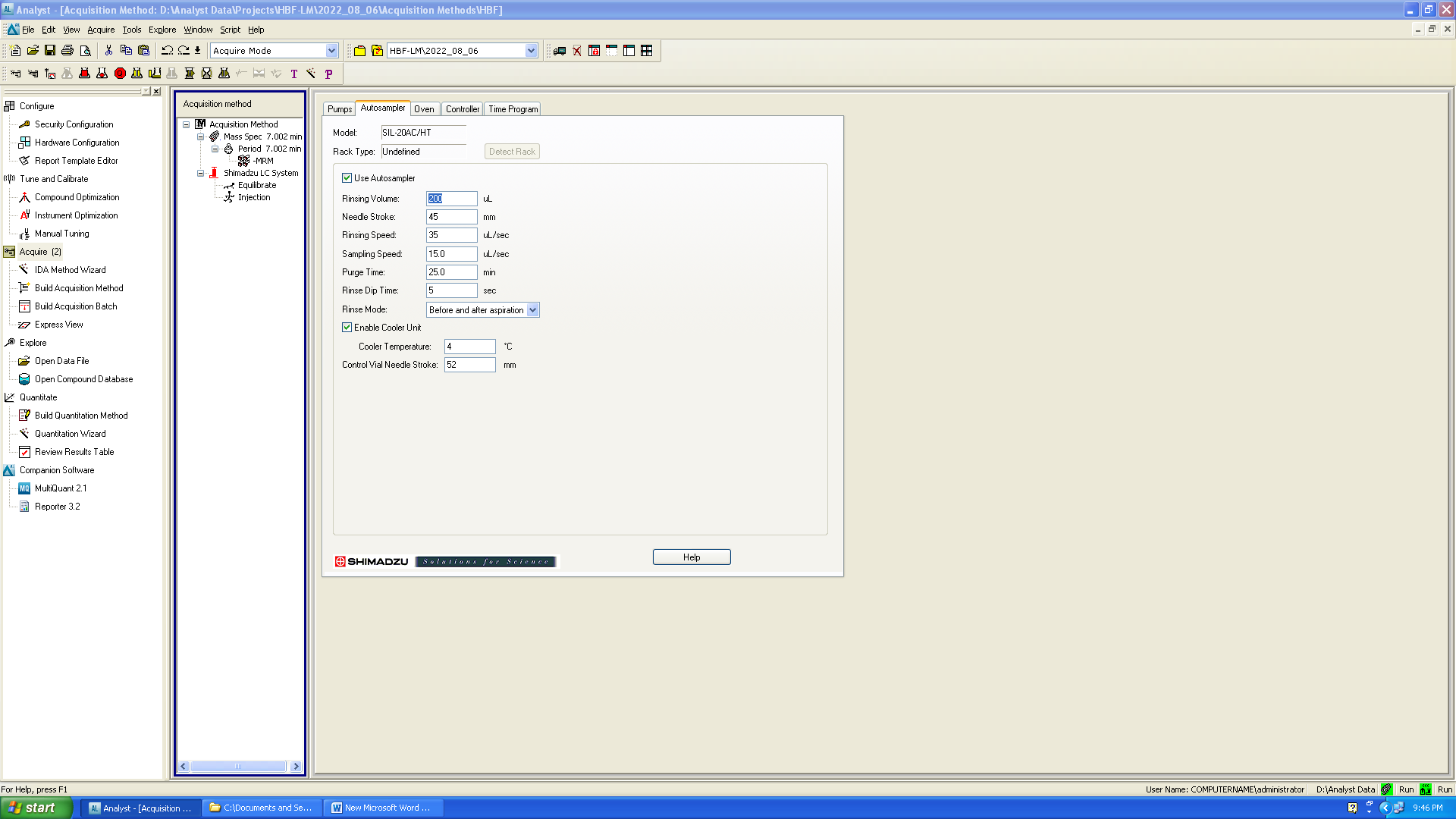

Supplement: S1 File — (DOCX) [file pone.0307995.s001.docx]
