# Supplementary material for: The effects of CYP2B6 inactivators on the metabolism of ciprofol
Source: PLoS One. 2024 Jul 29;19(7):e0307995. doi: 10.1371/journal.pone.0307995 (PMC11285948; doi:10.1371/journal.pone.0307995)
Supplement: Attachment — Submitted filename: Response to Reviewers.doc [file pone.0307995.s003.doc]

**Academic Editor:**

1. Please ensure that your manuscript meets PLOS ONE's style requirements, including those for file naming.

**Response:** Thank you for your reminder. We have modified the full text format and renamed the files according to the PLOS ONE style templates.

2. To comply with PLOS ONE submissions requirements, in your Methods section, please provide additional information regarding the experiments involving animals and ensure you have included details on (1) methods of sacrifice, (2) methods of anaesthesia and/or analgesia, and (3) efforts to alleviate suffering.

**Response:** Thank you very much for reminding us. We have added detailed information regarding the experiments involving animals to our Methods section.

3. Please state what role the funders took in the study. If the funders had no role, please state: "The funders had no role in study design, data collection and analysis, decision to publish, or preparation of the manuscript."

If this statement is not correct you must amend it as needed.

Please include this amended Role of Funder statement in your cover letter; we will change the online submission form on your behalf.

**Response:** We apologize for the confusion. In fact, the funders had no role in the study design, data collection and analysis, decision to publish, or preparation of the manuscript. We have amended the Role of Funder statement in our cover letter. On our behalf, please change the online submission form. Thanks!

4. Please confirm at this time whether or not your submission contains all raw data required to replicate the results of your study.

**Response:** All the raw data required to replicate the results of this study are presented in the Supporting Information files (named **“Raw Data”**) included in the attachment.

5. Your ethics statement should only appear in the Methods section of your manuscript. If your ethics statement is written in any section besides the Methods, please move it to the Methods section and delete it from any other section. Please ensure that your ethics statement is included in your manuscript, as the ethics statement entered into the online submission form will not be published alongside your manuscript.

**Response:** Thank you for your reminder. We have moved the ethics statement to the Methods section.

**Reviewers' comments:**

**Reviewer #1:**

1. Recently, in PMID: 37993078, the authors stated that the use of ciprofol in addition to CYP2B6 inhibitors might not be carried out any additional precaution in its use, dumping my enthusiasm in this report. However, I think that the authors need to explain much better what is good in their results and how this can complement the results reported in PMID: 37993078.；

**Response:** Thank you for your valuable suggestions. PMID: 37993078 shows that administering ciprofol for anaesthesia induction may not necessitate additional precautions in patients taking concomitant medications metabolized by CYP2B6 enzymes, such as acetaminophen, S-mephenytoin and bupropion. However, the results of our study suggest that CYP2B6 inactivators have the potential to inhibit the metabolism of ciprofol. This finding is a good complement to ciprofol-associated DDIs, and we have added relevant content to the Discussion section.

2. The authors also stated that the use of clopidogrel and psoralen might have a slightly longer duration of action for ciprofol. The authors must test this hypothesis in human liver microsomes at least. To complement the current literature.

**Response:** Thank you for your valuable suggestions. We have further validated the effects of psoralen and clopidogrel on the metabolism of ciprofol in human liver microsomes. The *IC*_50_ values of psoralen and clopidogrel were 34.05 μmol·L^-1^ and 2.565 μmol·L^-1^, respectively, showing mild inhibitory effects and moderate inhibitory effects on ciprofol metabolism, respectively. The effects of the two CYP2B6 inactivators on the metabolism of ciprofol in human liver microsomes were stronger than those in rat liver microsomes. We have added these results to the manuscript.

3. The English Language must be polished.

**Response:** Thank you for your reminder. The English language of the manuscript has been polished, and the editing certification has been uploaded as an attachment (named **“Editing Certification”**). However, in order not to interfere with your review, we have not highlighted individual changes in the manuscript that reflect English language polishing.

**Reviewer #2:**

1. When a patient is using two or more drugs simultaneously drug-drug interaction studies is to be evaluated. When patient will use this combination? Is it short term or long term treatment?

**Response:** Considering the indications for psoralen and clopidogrel, patients may be administered these drugs for a prolonged period of time, and DDIs need to be assessed if the patient receives ciprofol anaesthesia for surgical or investigative procedures during the treatment period.

To avoid confusion for the readers, we have added relevant content to the Discussion section.

2. In HPLC analysis, four degree (4C) centigrade used in autosampler. Justify

**Response:** We derived log information from devices and took a screenshot of the temperature setting screen of the autosampler to demonstrate the use of 4°C in an autosampler for HPLC-MS/MS analysis. The proof document has been uploaded as an attachment (named **“Temperature Setting in Autosampler”**)

3. HPLC chromatograms are not clear. Provide high quality chromatograms.

**Response:** Thank you for your reminder. We have reuploaded high-quality chromatograms (1200 dpi).

4. Discussion should be elaborated on CYP2B6 inhibitors though this enzyme has limited importance in drug metabolism.

**Response:** Thank you very much for the valuable suggestions. We have supplemented our discussion of CYP2B6 inhibitors by reviewing the relevant literature and comparing the results of our study with those of previous studies.
