# Supplementary material for: The effects of CYP2B6 inactivators on the metabolism of ciprofol
Source: PLoS One. 2024 Jul 29;19(7):e0307995. doi: 10.1371/journal.pone.0307995 (PMC11285948; doi:10.1371/journal.pone.0307995)
Supplement: Peer review history

# Peer review: Decision Letter 0

**DOI:** 10.1371/journal.pone.0307995.r001

**Authors:** Abhishek Kumar Singh

23 May 2024

PONE-D-24-14059The effects of CYP2B6 inactivators on the metabolism of ciprofolPLOS ONE

Dear Dr. Zhang,

Thank you for submitting your manuscript to PLOS ONE. After careful consideration, we feel that it has merit but does not fully meet PLOS ONE’s publication criteria as it currently stands. Therefore, we invite you to submit a revised version of the manuscript that addresses the points raised during the review process.

Please submit your revised manuscript by Jul 07 2024 11:59PM. If you will need more time than this to complete your revisions, please reply to this message or contact the journal office at plosone@plos.org. When you're ready to submit your revision, log on to https://www.editorialmanager.com/pone/ and select the 'Submissions Needing Revision' folder to locate your manuscript file.

Please include the following items when submitting your revised manuscript:

- A rebuttal letter that responds to each point raised by the academic editor and reviewer(s). You should upload this letter as a separate file labeled 'Response to Reviewers'.
- A marked-up copy of your manuscript that highlights changes made to the original version. You should upload this as a separate file labeled 'Revised Manuscript with Track Changes'.
- An unmarked version of your revised paper without tracked changes. You should upload this as a separate file labeled 'Manuscript'.

If you would like to make changes to your financial disclosure, please include your updated statement in your cover letter. Guidelines for resubmitting your figure files are available below the reviewer comments at the end of this letter.

If applicable, we recommend that you deposit your laboratory protocols in protocols.io to enhance the reproducibility of your results. Protocols.io assigns your protocol its own identifier (DOI) so that it can be cited independently in the future. For instructions see: https://journals.plos.org/plosone/s/submission-guidelines#loc-laboratory-protocols. Additionally, PLOS ONE offers an option for publishing peer-reviewed Lab Protocol articles, which describe protocols hosted on protocols.io. Read more information on sharing protocols at https://plos.org/protocols?utm_medium=editorial-email&utm_source=authorletters&utm_campaign=protocols.

We look forward to receiving your revised manuscript.

Kind regards,

Abhishek Kumar Singh, Ph.D.

Academic Editor

PLOS ONE

Journal Requirements:

When submitting your revision, we need you to address these additional requirements.

1. Please ensure that your manuscript meets PLOS ONE's style requirements, including those for file naming. The PLOS ONE style templates can be found at 

https://journals.plos.org/plosone/s/file?id=wjVg/PLOSOne_formatting_sample_main_body.pdf and 

https://journals.plos.org/plosone/s/file?id=ba62/PLOSOne_formatting_sample_title_authors_affiliations.pdf

2. To comply with PLOS ONE submissions requirements, in your Methods section, please provide additional information regarding the experiments involving animals and ensure you have included details on (1) methods of sacrifice, (2) methods of anesthesia and/or analgesia, and (3) efforts to alleviate suffering.

3. Thank you for stating the following financial disclosure: 

"This research was funded by the MEDICAL SCIENCE RESEARCH PROJECT OF HEBEI PROVINCE, grant number 20241993. "

Please state what role the funders took in the study.  If the funders had no role, please state: "The funders had no role in study design, data collection and analysis, decision to publish, or preparation of the manuscript." 

If this statement is not correct you must amend it as needed. 

Please include this amended Role of Funder statement in your cover letter; we will change the online submission form on your behalf.

4. We note that your Data Availability Statement is currently as follows: [All relevant data are within the manuscript and its Supporting Information files.]

Please confirm at this time whether or not your submission contains all raw data required to replicate the results of your study. Authors must share the “minimal data set” for their submission. PLOS defines the minimal data set to consist of the data required to replicate all study findings reported in the article, as well as related metadata and methods (https://journals.plos.org/plosone/s/data-availability#loc-minimal-data-set-definition).

For example, authors should submit the following data:

- The values behind the means, standard deviations and other measures reported;

- The values used to build graphs;

- The points extracted from images for analysis.

Authors do not need to submit their entire data set if only a portion of the data was used in the reported study.

If your submission does not contain these data, please either upload them as Supporting Information files or deposit them to a stable, public repository and provide us with the relevant URLs, DOIs, or accession numbers. For a list of recommended repositories, please see https://journals.plos.org/plosone/s/recommended-repositories.

If there are ethical or legal restrictions on sharing a de-identified data set, please explain them in detail (e.g., data contain potentially sensitive information, data are owned by a third-party organization, etc.) and who has imposed them (e.g., an ethics committee). Please also provide contact information for a data access committee, ethics committee, or other institutional body to which data requests may be sent. If data are owned by a third party, please indicate how others may request data access.

5. Your ethics statement should only appear in the Methods section of your manuscript. If your ethics statement is written in any section besides the Methods, please move it to the Methods section and delete it from any other section. Please ensure that your ethics statement is included in your manuscript, as the ethics statement entered into the online submission form will not be published alongside your manuscript. 

[Note: HTML markup is below. Please do not edit.]

Reviewers' comments:

Reviewer's Responses to Questions

**Comments to the Author**

1. Is the manuscript technically sound, and do the data support the conclusions?

The manuscript must describe a technically sound piece of scientific research with data that supports the conclusions. Experiments must have been conducted rigorously, with appropriate controls, replication, and sample sizes. The conclusions must be drawn appropriately based on the data presented. 

Reviewer #1: Yes

Reviewer #2: Yes

**********

2. Has the statistical analysis been performed appropriately and rigorously? 

Reviewer #1: Yes

Reviewer #2: Yes

**********

3. Have the authors made all data underlying the findings in their manuscript fully available?

The PLOS Data policy requires authors to make all data underlying the findings described in their manuscript fully available without restriction, with rare exception (please refer to the Data Availability Statement in the manuscript PDF file). The data should be provided as part of the manuscript or its supporting information, or deposited to a public repository. For example, in addition to summary statistics, the data points behind means, medians and variance measures should be available. If there are restrictions on publicly sharing data—e.g. participant privacy or use of data from a third party—those must be specified.

Reviewer #1: Yes

Reviewer #2: Yes

**********

4. Is the manuscript presented in an intelligible fashion and written in standard English?

PLOS ONE does not copyedit accepted manuscripts, so the language in submitted articles must be clear, correct, and unambiguous. Any typographical or grammatical errors should be corrected at revision, so please note any specific errors here.

Reviewer #1: Yes

Reviewer #2: Yes

**********

5. Review Comments to the Author

Please use the space provided to explain your answers to the questions above. You may also include additional comments for the author, including concerns about dual publication, research ethics, or publication ethics. (Please upload your review as an attachment if it exceeds 20,000 characters)

Reviewer #1: The authors described the potential drug-drug interaction of ciprofol, an anesthetic used in China and two CYP2B6 inhibitors, clopidogrel and psoralen. This was done due to the major enzyme responsible for the metabolism of ciprofol is CYP2B6.

the manuscript is written in a clear manner. but there are some points that I want to criticize. Recently, In PMID: 37993078 the authors stated that the use of ciprofol in addition to CYP2B6 inhibitors might not be carried out any additional precaution in its use, dumping my enthusiasm in this report. However, I think that the authors need to explain much better what is good in their results and how this can complement the results reported in PMID: 37993078.

The authors also stated that the use of clopidogrel and psoralen might have slightly long duration of action for ciprofol. The authors must test this hypothesis in human liver microsomes at least. To complement the current literature.

The English Language must be polished.

Major revisions are necessary.

Reviewer #2: The study is well designed and performed. But the following is to be addressed.

1. When a patient is using two or more drugs simultaneously drug-drug interaction studies is to be evaluated. When patient will use this combination? Is it short term or long term treatment?

2. In HPLC analysis, four degree (4C) centigrade used in autosampler. Justify

3. HPLC chromatograms are not clear. Provide high quality chromatograms

4. Discussion should be elaborated on CYP2B6 inhibitors though this enzymes have limited importance in drug metabolism.

**********

6. PLOS authors have the option to publish the peer review history of their article (what does this mean?). If published, this will include your full peer review and any attached files.

If you choose “no”, your identity will remain anonymous but your review may still be made public.

**Do you want your identity to be public for this peer review?** For information about this choice, including consent withdrawal, please see our Privacy Policy.

Reviewer #1: No

Reviewer #2: No

**********

[NOTE: If reviewer comments were submitted as an attachment file, they will be attached to this email and accessible via the submission site. Please log into your account, locate the manuscript record, and check for the action link "View Attachments". If this link does not appear, there are no attachment files.]

While revising your submission, please upload your figure files to the Preflight Analysis and Conversion Engine (PACE) digital diagnostic tool, https://pacev2.apexcovantage.com/. PACE helps ensure that figures meet PLOS requirements. To use PACE, you must first register as a user. Registration is free. Then, login and navigate to the UPLOAD tab, where you will find detailed instructions on how to use the tool. If you encounter any issues or have any questions when using PACE, please email PLOS at figures@plos.org. Please note that Supporting Information files do not need this step.

# Author comment: Author response to Decision Letter 0

**DOI:** 10.1371/journal.pone.0307995.r002

3 Jul 2024

Academic Editors' comments:

1. Please ensure that your manuscript meets PLOS ONE's style requirements, including those for file naming.

Response: Thank you for your reminder. We have modified the full text format and renamed the files according to the PLOS ONE style templates.

2. To comply with PLOS ONE submissions requirements, in your Methods section, please provide additional information regarding the experiments involving animals and ensure you have included details on (1) methods of sacrifice, (2) methods of anaesthesia and/or analgesia, and (3) efforts to alleviate suffering.

Response: Thank you very much for reminding us. We have added detailed information regarding the experiments involving animals to our Methods section.

3. Please state what role the funders took in the study. If the funders had no role, please state: "The funders had no role in study design, data collection and analysis, decision to publish, or preparation of the manuscript."

If this statement is not correct you must amend it as needed.

Please include this amended Role of Funder statement in your cover letter; we will change the online submission form on your behalf.

Response: We apologize for the confusion. In fact, the funders had no role in the study design, data collection and analysis, decision to publish, or preparation of the manuscript. We have amended the Role of Funder statement in our cover letter. On our behalf, please change the online submission form. Thanks!

4. Please confirm at this time whether or not your submission contains all raw data required to replicate the results of your study.

Response: All the raw data required to replicate the results of this study are presented in the Supporting Information files (named “Raw Data”) included in the attachment.

5. Your ethics statement should only appear in the Methods section of your manuscript. If your ethics statement is written in any section besides the Methods, please move it to the Methods section and delete it from any other section. Please ensure that your ethics statement is included in your manuscript, as the ethics statement entered into the online submission form will not be published alongside your manuscript.

Response: Thank you for your reminder. We have moved the ethics statement to the Methods section.

Reviewers' comments:

Reviewer #1:

1. Recently, in PMID: 37993078, the authors stated that the use of ciprofol in addition to CYP2B6 inhibitors might not be carried out any additional precaution in its use, dumping my enthusiasm in this report. However, I think that the authors need to explain much better what is good in their results and how this can complement the results reported in PMID: 37993078.；

Response: Thank you for your valuable suggestions. PMID: 37993078 shows that administering ciprofol for anaesthesia induction may not necessitate additional precautions in patients taking concomitant medications metabolized by CYP2B6 enzymes, such as acetaminophen, S-mephenytoin and bupropion. However, the results of our study suggest that CYP2B6 inactivators have the potential to inhibit the metabolism of ciprofol. This finding is a good complement to ciprofol-associated DDIs, and we have added relevant content to the Discussion section.

2. The authors also stated that the use of clopidogrel and psoralen might have a slightly longer duration of action for ciprofol. The authors must test this hypothesis in human liver microsomes at least. To complement the current literature.

Response: Thank you for your valuable suggestions. We have further validated the effects of psoralen and clopidogrel on the metabolism of ciprofol in human liver microsomes. The IC50 values of psoralen and clopidogrel were 34.05 μmol·L-1 and 2.565 μmol·L-1, respectively, showing mild inhibitory effects and moderate inhibitory effects on ciprofol metabolism, respectively. The effects of the two CYP2B6 inactivators on the metabolism of ciprofol in human liver microsomes were stronger than those in rat liver microsomes. We have added these results to the manuscript.

3. The English Language must be polished.

Response: Thank you for your reminder. The English language of the manuscript has been polished, and the editing certification has been uploaded as an attachment (named “Editing Certification”). However, in order not to interfere with your review, we have not highlighted individual changes in the manuscript that reflect English language polishing.

Reviewer #2:

1. When a patient is using two or more drugs simultaneously drug-drug interaction studies is to be evaluated. When patient will use this combination? Is it short term or long term treatment?

Response: Considering the indications for psoralen and clopidogrel, patients may be administered these drugs for a prolonged period of time, and DDIs need to be assessed if the patient receives ciprofol anaesthesia for surgical or investigative procedures during the treatment period.

To avoid confusion for the readers, we have added relevant content to the Discussion section.

2. In HPLC analysis, four degree (4C) centigrade used in autosampler. Justify

Response: We derived log information from devices and took a screenshot of the temperature setting screen of the autosampler to demonstrate the use of 4°C in an autosampler for HPLC-MS/MS analysis. The proof document has been uploaded as an attachment (named “Temperature Setting in Autosampler”)

3. HPLC chromatograms are not clear. Provide high quality chromatograms.

Response: Thank you for your reminder. We have reuploaded high-quality chromatograms (1200 dpi).

4. Discussion should be elaborated on CYP2B6 inhibitors though this enzyme has limited importance in drug metabolism.

Response: Thank you very much for the valuable suggestions. We have supplemented our discussion of CYP2B6 inhibitors by reviewing the relevant literature and comparing the results of our study with those of previous studies.

Attachment Submitted filename: Response to Reviewers.doc

# Editor report: Decision Letter 1

**DOI:** 10.1371/journal.pone.0307995.r003

**Authors:** Abhishek Kumar Singh

16 Jul 2024

The effects of CYP2B6 inactivators on the metabolism of ciprofol

PONE-D-24-14059R1

Dear Dr. Zhang,

We’re pleased to inform you that your manuscript has been judged scientifically suitable for publication and will be formally accepted for publication once it meets all outstanding technical requirements.

Within one week, you’ll receive an e-mail detailing the required amendments. When these have been addressed, you’ll receive a formal acceptance letter and your manuscript will be scheduled for publication.

An invoice will be generated when your article is formally accepted. Please note, if your institution has a publishing partnership with PLOS and your article meets the relevant criteria, all or part of your publication costs will be covered. Please make sure your user information is up-to-date by logging into Editorial Manager at Editorial Manager® and clicking the ‘Update My Information' link at the top of the page. If you have any questions relating to publication charges, please contact our Author Billing department directly at authorbilling@plos.org.

If your institution or institutions have a press office, please notify them about your upcoming paper to help maximize its impact. If they’ll be preparing press materials, please inform our press team as soon as possible -- no later than 48 hours after receiving the formal acceptance. Your manuscript will remain under strict press embargo until 2 pm Eastern Time on the date of publication. For more information, please contact onepress@plos.org.

Kind regards,

Abhishek Kumar Singh, Ph.D.

Academic Editor

PLOS ONE

Additional Editor Comments (optional):

Reviewers' comments:

# Editor report: Acceptance letter

**DOI:** 10.1371/journal.pone.0307995.r004

**Authors:** Abhishek Kumar Singh

19 Jul 2024

PONE-D-24-14059R1 

PLOS ONE

Dear Dr. Zhang, 

I'm pleased to inform you that your manuscript has been deemed suitable for publication in PLOS ONE. Congratulations! Your manuscript is now being handed over to our production team.

At this stage, our production department will prepare your paper for publication. This includes ensuring the following:

* All references, tables, and figures are properly cited

* All relevant supporting information is included in the manuscript submission,

* There are no issues that prevent the paper from being properly typeset

If revisions are needed, the production department will contact you directly to resolve them. If no revisions are needed, you will receive an email when the publication date has been set. At this time, we do not offer pre-publication proofs to authors during production of the accepted work. Please keep in mind that we are working through a large volume of accepted articles, so please give us a few weeks to review your paper and let you know the next and final steps. 

Lastly, if your institution or institutions have a press office, please let them know about your upcoming paper now to help maximize its impact. If they'll be preparing press materials, please inform our press team within the next 48 hours. Your manuscript will remain under strict press embargo until 2 pm Eastern Time on the date of publication. For more information, please contact onepress@plos.org.

If we can help with anything else, please email us at customercare@plos.org.

Thank you for submitting your work to PLOS ONE and supporting open access. 

Kind regards, 

PLOS ONE Editorial Office Staff

on behalf of

Dr. Abhishek Kumar Singh 

Academic Editor

PLOS ONE
